# Magnetically Functionalized Moss Biomass as Biosorbent for Efficient Co^2+^ Ions and Thioflavin T Removal

**DOI:** 10.3390/ma13163619

**Published:** 2020-08-16

**Authors:** Martin Pipíška, Simona Zarodňanská, Miroslav Horník, Libor Ďuriška, Marián Holub, Ivo Šafařík

**Affiliations:** 1 Department of Chemistry, Faculty of Education, Trnava University in Trnava, Priemyselná 4, P.O. Box 9, SK-918 43 Trnava, Slovakia; simona.zarodnanska@tvu.sk; 2 Department of Ecochemistry and Radioecology, Faculty of Natural Sciences, University of SS. Cyril and Methodius in Trnava, Nám. J. Herdu 2, SK-917 01 Trnava, Slovakia; miroslav.hornik@ucm.sk; 3 Institute of Materials Science, Faculty of Materials Science and Technology in Trnava, Slovak University of Technology in Bratislava, J. Bottu 25, SK-917 24 Trnava, Slovakia; libor.duriska@stuba.sk; 4 Institute of Environmental Engineering, Faculty of Civil Engineering, Technical University of Košice, Vysokoškolská 4, SK-042 00 Košice, Slovakia; marian.holub@tuke.sk; 5 Department of Nanobiotechnology, Biology Centre, ISB, CAS, Na Sádkách 7, 370 05 České Budějovice, Czech Republic; ivosaf@yahoo.com; 6 Regional Centre of Advanced Technologies and Materials, Palacky University, Šlechtitelů 27, 783 71 Olomouc, Czech Republic

**Keywords:** magnetic biosorbent, microwave synthesis, cobalt, thioflavin T, biosorption, reusability

## Abstract

Microwave synthesized iron oxide nanoparticles and microparticles were used to prepare a magnetically responsive biosorbent from *Rhytidiadelphus squarrosus* moss for the rapid and efficient removal of Co^2+^ ions and thioflavin T (TT). The biocomposite was extensively characterized using Fourier transformed infrared (FTIR), XRD, SEM, and EDX techniques. The magnetic biocomposite showed very good adsorption properties toward Co^2+^ ions and TT e.g., rapid kinetics, high adsorption capacity (218 μmol g^−1^ for Co and 483 μmol g^−1^ for TT), fast magnetic separation, and good reusability in four successive adsorption–desorption cycles. Besides the electrostatic attraction between the oxygen functional moieties of the biomass surface and both Co^2+^ and TT ions, synergistic interaction with the –FeOH groups of iron oxides also participates in adsorption. The obtained results indicate that the magnetically responsive biocomposite can be a suitable, easily separable, and recyclable biosorbent for water purification.

## 1. Introduction

Considerable efforts are currently being made to eliminate contaminants that have caused growing concern from the large volume of industrial effluents and wastewaters [1]. Among all the available conventional and advanced treatment processes, adsorption is one of the top treatment procedures for the effective removal of dissolved pollutants from the contaminated streams using appropriate adsorbents [2]. The constantly increasing the number of research papers on the biosorption of both organic and inorganic pollutants and radionuclides by various biological materials indicates that there is still concern regarding conventional adsorption, which researchers argue ought to be replaced with a more environmentally friendly approach (see e.g., [3]). Comprehensive reviews summarizing the current progress in the application of biosorption technology [4] and suggesting future trends for the efficient removal of pollutants [5,6] have been recently published. Despite superlative adsorption properties and the excellent performance of various biomass-based biosorbents, there are still obstacles that restrain the commercialization of biosorption. For instance, the complicated separation of exhausted biosorbents from water bodies limits their recyclability [7]. Generally, non-reusability is cost-ineffective and may restrict the application of biosorbents on a large scale. On the contrary, the application of reusable biosorbents minimizes the total amount of waste generation and reduces the process costs. 

Magnetic separation is a renewable recycling technology [8] that is characterized by higher efficiency compared with other separation methods (e.g., sedimentation, filtration) and is frequently applied in water purification. Magnetically responsive low-cost biological materials have been prepared using various modification procedures and subsequently used as biosorbents [2,9]. In addition to effective magnetic separation, magnetic functionalization may affect the adsorption capacity of biosorbents. However, contradictory findings related to the adsorption capacity of biosorbents after magnetic modification were recently published. The decrease of metals ions’ biosorption efficiency after magnetic modification is probably due to the blocking of binding sites on a biomass surface with deposited iron oxides [10]. Daneshfozoun et al. [11] revealed that the magnetic nanoparticles on the biosorbent surface could have enhanced the number of active sites, and the magnetic sorbent exhibited 10% higher Pb (II) removal efficiency than the unmodified counterpart. Liu et al. [8] described the synergistic adsorption effect during rhodamine B and methyl orange adsorption by Fe_3_O_4_ functionalized activated carbon, confirming the role of Fe_3_O_4_ nanoparticles in the removal of dyes. Specifically, magnetic modification may decrease the BET surface due to the formation of iron (hydro) oxides on sorbent surface, and some pollutants can be adsorbed more efficiently on the surfaces of these precipitated (hydro)oxides [12]. Surprisingly, the regeneration and reusability of magnetic biosorbent have not been realized in many studies [13,14,15], preventing assessment of the adsorption capacity and stability of biosorbents as well as magnetic separation efficiency during repetitive adsorption–desorption cycles. Therefore, further research on the magnetically responsive biomass adsorption performance of both organic and inorganic pollutants is of great importance to provide an insight into the biosorption mechanism and to ensure the applicability of biosorption technology for industrial effluents. 

Herein, the magnetic modification of moss *Rhytidiadelphus squarrosus* using microwave synthetized iron oxide nanoparticles and microparticles was realized. Prepared magnetic biosorbent was characterized (Fourier transformed infrared (FTIR), XRD, SEM, and EDX elemental mapping) and utilized for the adsorption of both metal ions and synthetic organic dye from aqueous solutions, and the effects of various environmental parameters on the adsorption performance were systematically investigated by batch adsorption experiments. Furthermore, the reusability and stability of magnetically modified biomass were evaluated. Thus, magnetically responsive biosorbent can be useful as an inexpensive material for the efficient separation of both organic dyes and metal ions from industrial effluents. In this work, cationic benzothiazole dye thioflavin T (TT) and Co^2+^ ions were used as models of organic dye and heavy metal, respectively. TT belongs to the cationic methine dyes, the most important group of methine dyes, which are used on a large scale in the textile industry to dye polyacrylonitrile fibers. Cobalt ions are present in the wastewaters of many industries; however, unlike other heavy metals such as Cd, Pb, Hg, Cu, and Zn, less attention is paid to Co removal processes from contaminated effluents.

## 2. Materials and Methods

### 2.1. Biomass Preparation

The biomass of *Rhytidiadelphus squarrosus* moss was collected from the spruce forests (49.1585° N, 20.2249° E) in High Tatras Mountains, Slovakia. The biomass was cleaned of macroinpurities, thoroughly washed in deionized water (Millipore, 0.054 µS cm^−1^), and oven-dried for 72 h at a maximum temperature of 60 °C to avoid the degradation of binding sites. Subsequently, the moss biomass was milled, sieved, and for magnetic modification, a fraction of 300–600 μm was applied. 

### 2.2. Magnetic Biosorbent Preparation

*R. squarrosus* biomass was magnetically modified according to the procedure of Safarik and Safarikova [16] using microwave-synthesized magnetic iron oxide nanoparticles and microparticles. Briefly, 1 g of FeSO_4_⋅7H_2_O was dissolved in 200 mL of water in a 1000 mL beaker, and 1 mol L^−1^ NaOH solution was slowly added under stirring until the pH reached the value of 12. Subsequently, the suspension was placed into a standard domestic microwave oven (700 W, 2.45 GHz) and treated for 10 min at the maximum power. Formed magnetic iron oxide particles were repeatedly washed with water to remove completely the remaining NaOH. Subsequently, 1 g of *R. squarrosus* biomass was thoroughly mixed with 2 mL of iron oxide suspension in water (1 part of completely sedimented iron oxide particles and 4 parts of water) to ensure a uniform distribution of magnetic nanoparticles and microparticles on biomass surfaces; a small amount of water can be added to facilitate the mixing process. Then, the magnetically modified biomass was dried at 60 °C for 24 h and used in adsorption experiments.

### 2.3. Adsorption of Co^2+^ Ions and Thioflavin T

CoCl_2_ and thioflavin T solutions (both analytical grade) were prepared using deionized water. Working thioflavin T solutions were obtained by diluting the stock solution (2505 μmol L^−1^). Adsorption kinetics trials were conducted by the addition of magnetic biosorbent (suspension density 2.5 g L^−1^) to Co (500 and 1000 μmol L^−1^) or thioflavin T (313 and 626 μmol L^−1^) solutions (pH 6.0). All Co solutions were spiked with ^60^CoCl_2_ (37.5 kBq L^−1^). Vials were shaken on a rotary mixer (45 rpm) at 22 °C and at time intervals (5–1440 min), the biosorbent was magnetically separated for 30 s and scintillation gamma spectrometry was used to measure ^60^Co radioactivity in supernatant fluids. In the case of thioflavin T, the absorbance of supernatants was measured by a Varian Cary 50 UV-Vis spectrophotometer (Varian, Mulgrave, Australia) at 413 nm. Co^2+^ and thioflavin T quantities *Q_eq_* (μmol g^−1^) separated by magnetic biosorbent were calculated as follows:

(1) $$Q_{t}=\left(C_{0}-C_{t}\right)*V/M$$

where *C*_0_ is the initial concentration and *C_t_* is the solution concentration of thioflavin T and Co^2+^ at time *t* (μmol L^−1^), *M* represents magnetic biosorbent dosage (g), and *V* is the total solution volume (L).

The adsorption capacity of magnetic biosorbent (suspension density 2.5 g L^−1^) was analyzed in solutions at initial thioflavin T concentrations (155–2505 μmol L^−1^) and CoCl_2_ concentrations ranging from 100 to 4000 μmol L^−1^ spiked with ^60^CoCl_2_. Vials were shaken on a rotary mixer (45 rpm) at 22 °C for 4 h. At the end of the experiments after biosorbent separation, the radioactivity or absorbance of the supernatants was measured. Calculations of adsorption capacities *Q_eq_* (μmol g^−1^) were according to the following equation:

(2) $$Q_{eq}=\left(C_{0}-C_{eq}\right)*V/M$$

where *C*_0_ is the initial concentration and *C_eq_* is the equilibrium concentration of thioflavin T or Co^2+^ (μmol L^−1^), *M* represents the magnetic biosorbent dosage (g), and *V* represents the total solution volume (L).

To determine the effect of pH on adsorption, the magnetic biosorbent was shaken (4 h at 22 °C) in Co^2+^ (C_0_ = 1000 μM) and thioflavin T solutions (C_0_ = 313 μM) of desired pH (2.0–8.0) adjusted by adding 0.1 M HCl or 0.1 M NaOH. All experiments were performed in duplicate. 

MINEQL+ ver. 4.5 was used for the prediction of Co speciation in the adsorption systems as a function of solution pH, concentration of cations and anions, ionic strength, and temperature.

### 2.4. Magnetic Biosorbent Reusability and Stability

The stability and reusability of magnetic biosorbent were determined through 4 successive adsorption–desorption cycles. The desorption of thioflavin T and Co^2+^ ions from magnetic biosorbent was realized using 0.1 M acetic acid or 0.1 M HCl, respectively as extraction solutions. Desorption was carried out on a rotary mixer (45 rpm) for 4 h at 22 °C and a liquid/solid (L/S) ratio 0.4 and 0.8 L/g, respectively. After desorption, the biosorbent was magnetically separated, repeatedly washed with deionized water, and used in the next adsorption–desorption cycle. 

### 2.5. SEM-EDX, FTIR, and XRD Analyses

The Fourier transformed infrared (FTIR) absorption spectra of native and magnetically modified biomass of *R. squarrosus* were determined using an ATR modul (Nicolet iS50, Thermo Fisher Scientific, Waltham, MA, USA) in the mid-infrared (4000–400 cm^−1^) region. The surface structure morphology and microstructure of native biomass and magnetic biosorbent before and after Co^2+^ adsorption and energy-dispersive X-ray spectroscopy were realized by JEOL JSM7600F (Mitaka, Tokyo, Japan) and VEGA 3 SEM (TESCAN s.r.o., Brno, Czech Republic) microscopes. Magnetic biosorbent samples, oven-dried at 60 °C for 24 h, were coated with Au before SEM-EDX analysis. X-ray diffraction (XRD) analysis was performed using a Philips PW 1830 diffractometer with iron-filtered Co Kα_1,2_ radiation to identify the crystalline structure of magnetic nanoparticles and microparticles deposited on biomass surfaces. The XRD patterns were recorded in the 2θ range between 10° and 80° with a 0.02° step size and an exposure time of 10 s per step.

### 2.6. Radiometric Analysis

Standardized ^60^CoCl_2_ solution (5.181 MBq mL^−1^, CoCl_2_ 20 mg L^−1^ in 3 g L^−1^ HCl) was obtained from the Czech Institute of Metrology, Prague (Czech Republic). A gamma-spectrometric scintillation detector 54BP54/2-X (Scionix, LA Bunnik, The Netherlands) with a well-type NaI (Tl) crystal and Ortec ScintiVision-32 software (ORTEC, Oak Ridge, TN, USA) were applied for the radioanalysis of ^60^Co in magnetic moss biosorbent and supernatant fluids. A typical γ-ray peak for ^60^Co (Eγ = 1173.24 keV) was selected for efficiency and energy calibration. ^60^CoCl_2_ (^60^Co T_1/2_ = 5.3 y) standard solution with known radioactivity was applied for calibration.

## 3. Results and Discussion

### 3.1. Magnetic Biosorbent Characterization

**Figure 1:**
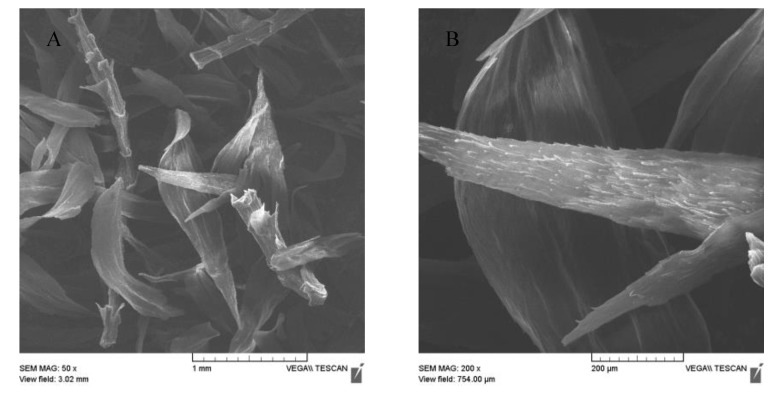
SEM images of native biomass of moss *R. squarrosus* (**A**) magnification 50×, (**B**) 200×.

**Figure 2:**
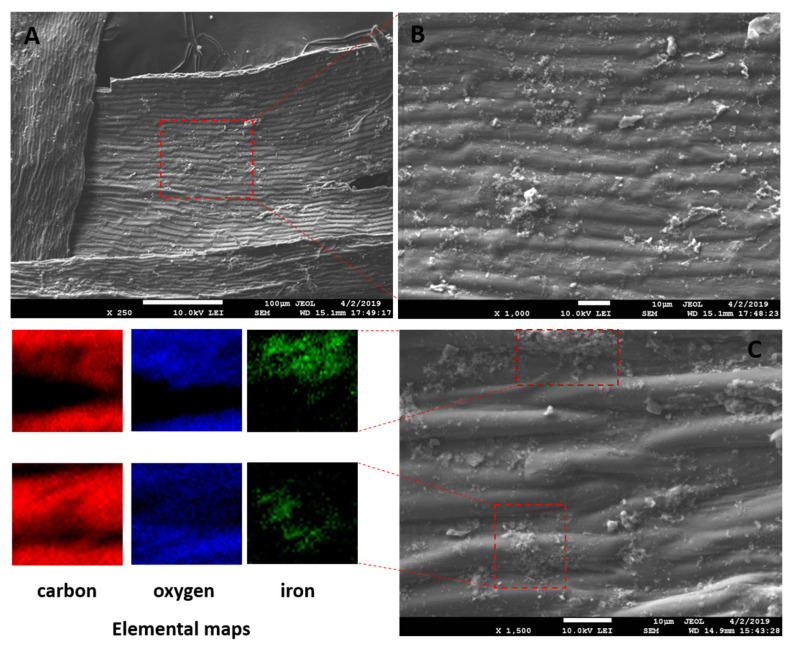
SEM and EDX images of magnetic responsive biomass of *R. squarrosus*. (**A**) Magnification 250×, (**B**) 1000×, (**C**) 1500×. The SEM image in panel B represents the area closed by the red dashed line in panel A. EDX elemental distribution maps show the elemental (C, O and Fe) composition of areas closed by red dashed lines in panel C.

The magnetization procedure used for *R. squarrosus* modification is cost-effective and very simple, utilizing biomass contact with a suspension of microwave-synthesized nanoparticles and microparticles of iron oxide in water [2]. The SEM images in Figure 1 and Figure 2 show the morphology and surface structure of the native moss biomass and magnetic biocomposite. Pristine biomass is characterized by a relatively smooth surface, tiny wrinkles, and without the presence of any particulate matter on the moss leaves (Figure 1A,B). After magnetic modification (Figure 2), microparticles and micrometer-sized aggregates with a good dispersion on biomass surfaces are present (highlighted by red dashed line, Figure 2C). Detailed EDX elemental analysis revealed that the deposited fine particles and aggregates are rich in Fe and O but comparatively low in carbon, which we interpreted to be iron oxide particles (Figure 2C).

**Figure 3:**
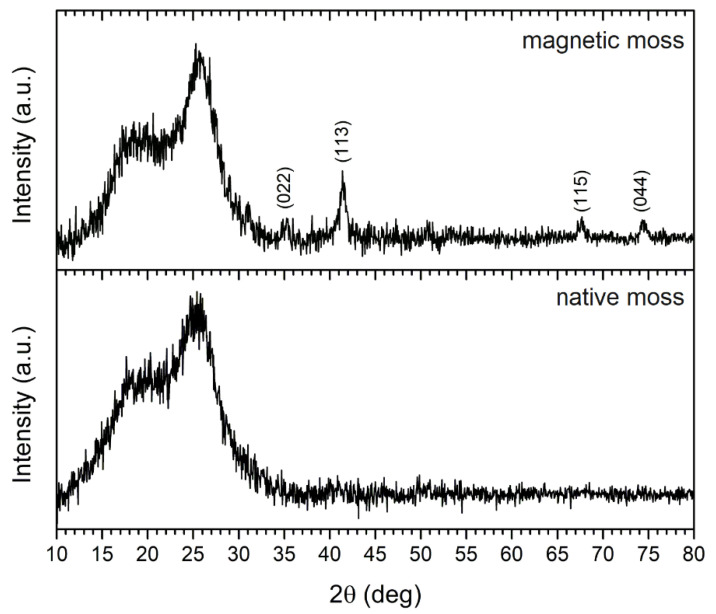
XRD pattern of native and magnetically modified *R. squarrosus* recorded using Co Kα_1,2_ radiation.

XRD analysis was applied to identify the crystalline structure of the magnetic biocomposite, and the X-ray diffraction pattern of the native and magnetic biosorbent is shown in Figure 3. The degree of crystallinity of moss biomass at 15–18° and 26° is reported to be due to cellulosic polymorphs [10]. The intense diffraction peaks observed at 2θ values of 35.3°, 41.4°, 67.7°, and 74.6° were indexed to (022), (113), (115), and (044) crystallographic planes, respectively, which indicates the presence of the standard face-centered cubic Fe_3_O_4_ (magnetite) on magnetically modified biomass surfaces. In previous work, where the same magnetization method was employed [17], Mössbauer spectroscopy confirmed the presence of non-stoichiometric magnetite in stable aggregates. Moreover, the crystalline size *L* (nm) of Fe_3_O_4_ on the biomass surface was determined using the Scherrer equation:

(3) $$L=\frac{K\times \lambda }{\beta \times cos\theta }$$

where λ is the X-ray wavelength (nm), *β* is the FWHM (full width at half maximum) of the peak (rad), *K* is a dimensionless shape factor that varies with the crystal shape (0.94), and *θ* is the diffraction angle (rad). The average particle size of Fe_3_O_4_ organized into micrometer-sized aggregates on the biomass surface calculated from diffraction peaks (see above) was ca 13 nm, which is consistent with previous results [2]. It is important to note that upon exposure to O atoms, magnetite oxidizes to maghemite with the partial conversion of Fe^2+^ ions into Fe^3+^ ions [18]. The as-synthetized iron oxide particles exhibited a good dispersibility on the biomass surface (Figure 2B), and thus, magnetically modified *R. squarrosus* shows a rapid magnetic response to an external magnetic field, enabling a rapid and selective separation of the biosorbent from aqueous solutions using a permanent magnet [19].

**Figure 4:**
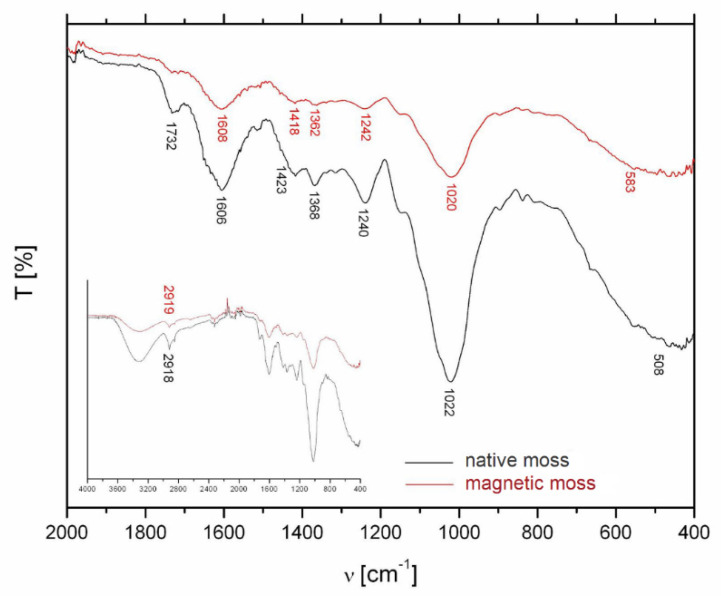
FTIR spectra of *R. squarrosus* biomass before and after magnetic modification. Inset: the whole FTIR spectrum in mid-infrared region.

The pristine and magnetically modified biomass was characterized by spectral analysis in the mid-infrared region (4000–400 cm^−1^) to identify major functional groups (Figure 4). In the FTIR spectrum of native biomass, the wide band at 3400–3200 cm^−1^ could be assigned to the vibrational modes of hydroxyl groups. The sharp peak at 2919 cm^−1^ corresponds to the asymmetric stretching vibration of C–H in the biopolymers (polysaccharides, lipids) (Figure 4, inset). The band at 1732 cm^−1^ is caused by symmetric stretching vibrations of C=O in esters, while the bands at 1606 and 1423 cm^−1^ correspond to the strong asymmetric and weak symmetric stretching of the carboxylate ion (COO^−^) [20,21]. Intensity of these bands decreased after the magnetic modification (Figure 4). The absorption bands at 1240 and 1242 cm^−1^ correspond to the asymmetric vibration of PO_2_^−^ of nucleic acids and the peaks at 1022 and 1020 cm^−1^ could be attributed to the C–O stretch in oligosaccharides, glycoprotein, and cellulose [22]. We can summarize that both samples exhibit similar patterns with band intensity changes and minor band positions shifts. These were caused by a slight effect of deposited iron oxide particles on the surface of the moss biomass.

### 3.2. Thioflavin T and Co^2+^ Ions Adsorption

A prepared magnetically responsive biosorbent was applied for the adsorption of both cationic benzothiazole dye thioflavin T (TT) and Co^2+^ ions. To obtain precise quantitative data, characterizing the adsorption of Co^2+^ ions by the magnetic biosorbent radiotracer technique with ^60^Co as a radioindicator was employed. 

**Figure 5:**
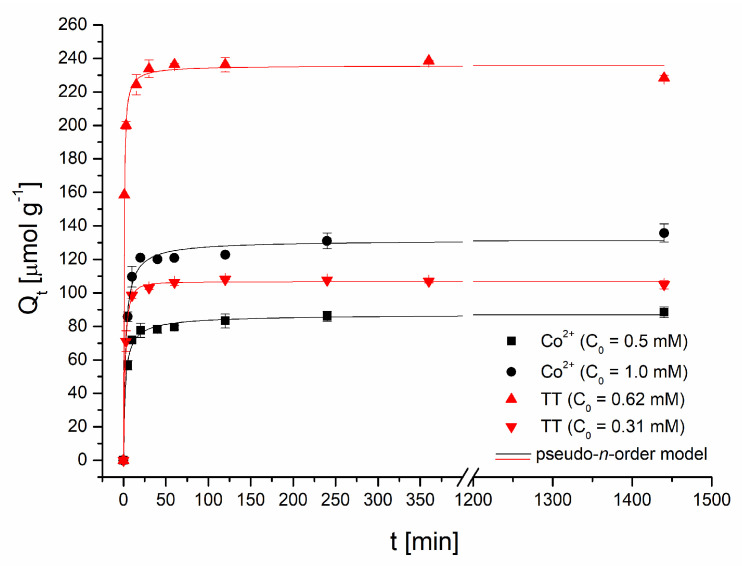
Effect of the reaction time on Co^2+^ and thioflavin T adsorption by magnetically responsive biosorbent (2.5 g L^−1^; T = 22 °C; pH 6.0).

*Adsorption kinetics.* The time dependence of TT and Co^2+^ ions uptake is presented in Figure 5. Thioflavin T adsorption was fast (Figure 5); the adsorption capacity of the magnetic biosorbent rapidly increased, and after 30 min, it reached 102.9 μmol g^−1^ (C_0_ = 0.31 mM) and 233.8 μmol g^−1^ (C_0_ = 0.62 mM) as readily available surface binding sites were occupied. Equilibrium was achieved at approximately 60 min and the TT adsorption capacity reached 106.4 and 236.4 μmol g^−1^, respectively. A nearly identical kinetic profile for TT was detected when native *R. squarrosus* biomass was used [23]. A slightly different pattern was observed during Co adsorption. In the first phase (60 min) similarly to TT kinetics, the adsorption capacity rose rapidly to 79.4 μmol g^−1^ (C_0_ = 0.5 mM) and 120.9 μmol g^−1^ (C_0_ = 1 mM) as a result of a higher driving force. The second phase is characterized by a slower increase in adsorption capacity (88.5 and 135.8 μmol g^−1^), and the equilibrium state was reached at 240 min. When native biomass was used, equilibrium was achieved faster: after only 60 min [24]. Similar results were reported for Pb^2+^ and Cd^2+^ biosorption by magnetic *Bauhinuia purpurea* pods [10]. Lei et al. [25] revealed that Fe_3_O_4_ magnetic nanoparticles exhibited a more rapid adsorption rate in the initial phase of Cd^2+^ adsorption (before 90 min) and then, similarly as in our experiments, the adsorption rate tends to be flat. The observed distinctions in the kinetics profiles of organic dye and metal ions are caused by (1) different mechanisms of Co^2+^ and TT adsorption, and (2) the distinct affinity of Co^2+^ and thioflavin T to binding sites on magnetically responsive biosorbent.

To quantify the initial rapid adsorption of both Co^2+^ and TT, kinetic data were analyzed using a pseudo-*n*-order kinetic model derived from the general rate equation [26], which can be described as follows:

(4) $$Q_{t}=\;Q_{e}-{\left(Q_{e}^{1-n}-\;k_{n}t\;(1-n)\right)}^{1/\left(1-n\right)}$$

where *Q_e_* and *Q_t_* are the quantity of Co and TT (μmol g^−1^) adsorbed at equilibrium and at time *t* (min), respectively, *k_n_* is the pseudo-*n*-order rate constant ((min^−1^) (mg g^−1^)^1−n^), and *n* is the order of adsorption with respect to the effective concentration of binding sites on the biosorbent surface [8]. It is apparent from Table 1 that both kinetics data were well fitted with the pseudo-*n*-order kinetic model with high *R*^2^ values and a high comparability of *Q_eqcal_* to the experimental values of *Q_eqexp_*. The adsorption rate values (*r*) that were calculated using the equation

(5) $$r=k_{n}Q_{e}^{\;\;\;n}$$

gradually rose, with the increasing initial concentrations of Co^2+^ and TT in the adsorption system indicating faster removal at higher Co^2+^ and TT concentrations. Moreover, the fractional numbers *n* were close to 2, implying that the determining step of the biosorption of both Co^2+^ and TT by magnetically responsive biomass is chemical adsorption. Similarly, the order of adsorption for rhodamine B and methyl orange removal by Fe_3_O_4_ nanoparticles-functionalized activated carbon obtained from the pseudo-*n*-order model varied from 1 to 2 and was closer to 2 [8]. On the contrary, Fraga et al. [27] observed the great value of *n* (7.46) for the adsorption of reactive drimaren red by amino-Fe_3_O_4_ functionalized multilayer graphene oxide, suggesting a highly heterogeneous interface.

*Adsorption equilibrium.* To determine the cobalt and thioflavin T removal capacities by magnetically responsive biomass, isotherm trials were carried out in batch mode (C_0_ TT 0.16–2.51 mmol L^−1^; C_0_ Co^2+^ 0.1–4 mmol L^−1^, biosorbent dosage = 2.5 g L^−1^; pH 6.0; time = 4 h; T = 22 °C). Isotherm models of Langmuir (6) and Freundlich (7) were employed to provide quantitative information about Co^2+^ and TT adsorption. The nonlinear forms are expressed as follows:

(6) $$Q_{eq}=\frac{bQ_{max}C_{eq}}{1+bC_{eq}}$$

(7) $$Q_{eq}=KC_{eq}^{\;\;\left(1/n\right)}$$

where *b* (L μmol^−1^) is constant related to the affinity of biosorbent binding sites and *Q_max_* (μmol g^−1^) is a Langmuir constant which indicates the maximum adsorption capacity of biosorbent with monolayer surface coverage. *K* [(µmol g^−1^) (L µmol^−1^)^1/*n*^] is a Freundlich constant referring to the adsorption capacity, and 1*/n* is a dimensionless unit representing surface heterogeneity.

**Figure 6:**
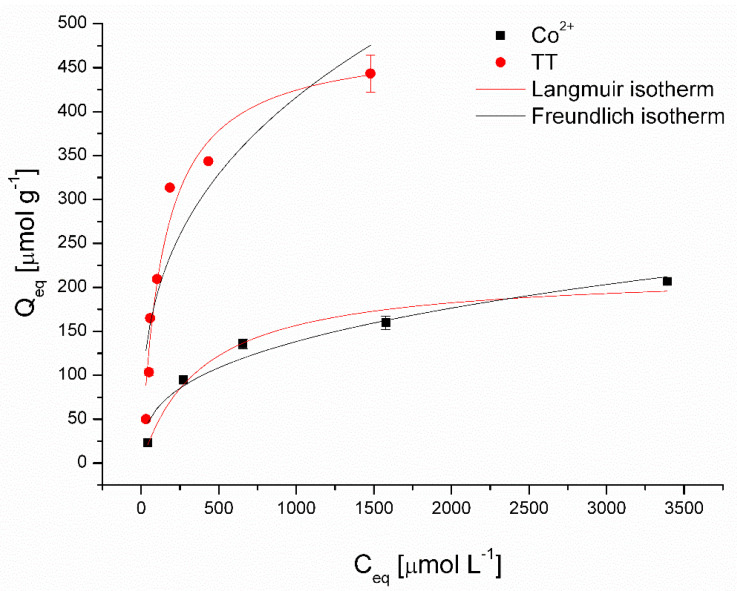
Langmuir and Freundlich isotherm models for Co^2+^ and thioflavin T adsorption by magnetically responsive biosorbents (2.5 g L^−1^; T = 22 °C; pH 6.0).

Figure 6 presents the nonlinear fitting results of the Langmuir and Freundlich models, and Table 2 summarizes the calculated isotherm parameters for the removal of Co^2+^ and TT using magnetically responsive biomass as the adsorbent. The higher coefficients of determination (*R*^2^) of the Langmuir model for both Co (0.980) and TT (0.961) removal suggested that the adsorption behavior could be attributed to the monolayer chemical adsorption mechanisms [28]. Moreover, reflecting values of affinity constant *b*, the magnetic biosorbent exhibited considerably higher affinity for TT (0.0072 L μmol^−1^) compared to Co^2+^ ions (0.0025 L μmol^−1^). The maximum adsorption capacity *Q_max_* at 22 °C was 483 µmol g^−1^ for TT and 218 µmol g^−1^ for Co^2+^ ions, respectively. It should be highlighted that the *Q_max_* value of the magnetic biosorbent for Co^2+^ ions is slightly higher than that of the native biomass counterpart, and for TT, it is significantly higher (Table 2). Improved adsorption capacity, especially in the case of TT removal, could be ascribed to biomass modification, which led not only to selective magnetic biosorbent separation but also to a synergistic adsorption effect (see discussion below). Such a synergistic effect in methyl orange and rhodamine B removal after the Fe_3_O_4_ functionalization of activated carbon was observed by Liu et al. [8]. 

Compared with the other reported organic and inorganic adsorbents (Table 3), the adsorption capacity of TT onto the magnetically responsive biosorbents showed a better performance. The adsorption capacity of Co^2+^ ions is higher than commercial magnetite and comparable with magnetic nanocomposites; however, it is significantly lower than that of the algae magnetic composite (Table 3). Such results confirmed the feasibility of employing magnetic biomass as adsorbents for both dye and metal ions. Moreover, the magnetic biosorbent is superior to other adsorbents in terms of its extremely simple preparation, efficient separation, and reusable properties (see discussion below). 

**Figure 7:**
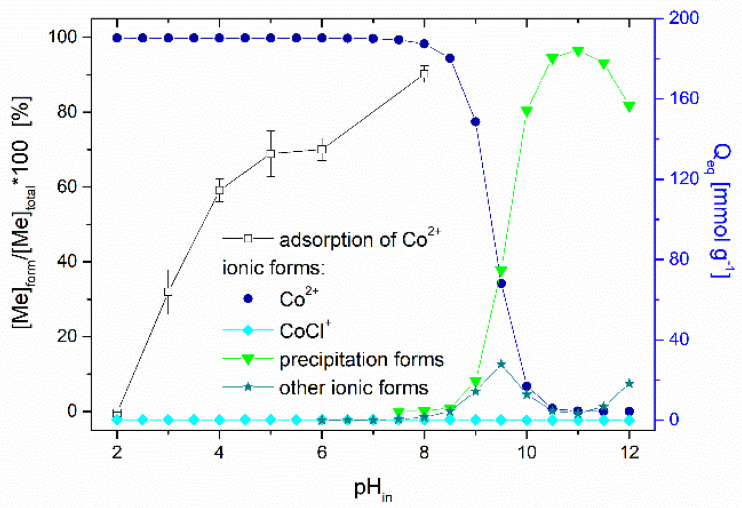
Effect of initial pH on the adsorption of Co (1000 µmol L^−1^ CoCl_2_, ^60^CoCl_2_ 75 kBq L^−1^) by magnetically responsive biosorbent (2.5 g L^−1^; T = 22 °C). Co speciation calculated using MINEQL+ ver. 4.5, initial conditions: 1000 µmol L^−1^ CoCl_2_, pCO_2_ = 38.5 Pa, T = 22 °C. The solution pH after sorption increased from the initial values of 2.0, 3.0, 4.0, 5.0, 6.0 and 8.0 to 2.4, 4.3, 5.1, 5.3, 5.9, and 7.2.

*Effect of pH.* The solution pH affects the ionization and speciation of both Co ions and thioflavin T molecules, as well as the dissociation of oxygen-containing functional groups (carboxyl and hydroxyl) on the biosorbent surface. The pH_pzc_ of the magnetically responsive biosorbent determined by the pH drift method was 5.4. Figure 7 presents the impact of the initial solution pH on the adsorption performance of Co^2+^ ions by the magnetic biosorbent. As was reported in our previous work [24], the same curve is also characteristic for Co^2+^ adsorption by native *R. squarrosus* biomass. The adsorption capacity gradually increased from 3.6 to 134.8 µmol g^−1^ with the pH increasing from 2.0 to 6.0. The negligible uptake of Co ions observed at pH 2.0 could be closely related to the protonation of the oxygen-containing functional moieties (e.g., –COOH) of the biosorbent surface, resulting in a competition (or ion exchange) between cobalt ions and the H^+^ or H_3_O^+^ ions in solution and electrostatic repulsion between the positively charged biomass surface and Co^2+^ ions. As the pH increased, the competition between Co^2+^ and H^+^ for functional moieties reduced significantly. Considering the pH_pzc_ value, the biosorbent surface was negatively charged at >pH 5.4, and the electrostatic attraction between O-containing functional moieties and Co^2+^ ions became stronger, resulting in higher adsorption capacity (Figure 7). Specifically, glucuronic and galacturonic acids are more abundant in the primary cell walls of mosses than in vascular plants [39], suggesting the relevance of their –COO^−^ groups in the Co removal capacity. 

The speciation of cobalt calculated using the MINEQL+ program indicated that Co particularly occurs as a Co^2+^ (99.7%) cation within pH ranging from 2.0 to 8.0 (Figure 7). Other cobalt ionic forms (Co(OH)_4_^4+^, Co(OH)^3−^) are present at a solution pH > 8.0, and Co precipitation begins at pH > 8.0. We suppose that the notably higher removal capacity (172.3 µmol g^−1^) at the initial pH of 8.0 was related also to the formation of Co(OH)_2_ precipitates directly on biosorbent surfaces.

**Figure 8:**
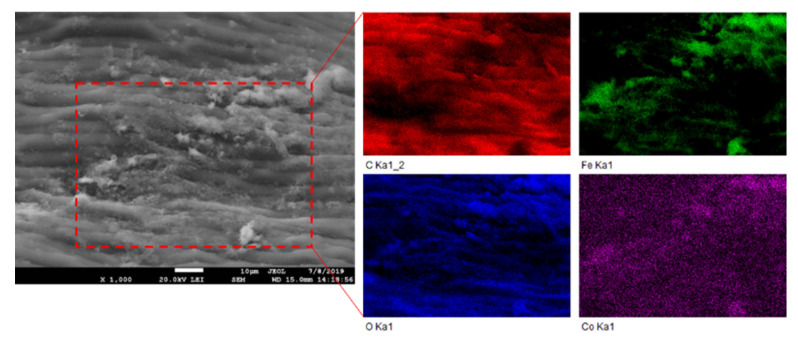
SEM image and EDX elemental maps of magnetically responsive biomass after Co^2+^ adsorption. The elemental maps show C, Fe, O, and Co concentrations in an area enclosed by red dashed lines.

Based on the comparison of *Q_max_* values of native and magnetically modified biomass for Co (Table 2), we assume that in addition to the O-functional moieties of biopolymers, the Fe_3_O_4_ microparticles and aggregates deposited on the biomass surfaces also contribute to Co^2+^ adsorption. Therefore, EDX elemental mapping results were evaluated to clarify the role of Fe_3_O_4_ in Co adsorption by the magnetic biosorbent (Figure 8). It is evident that the distribution of Co on the biomass surface is closely associated with almost all Fe_3_O_4_ aggregates (Fe and O-rich regions on elemental maps, Figure 8), indicating that the synergistic interactions with Fe_3_O_4_ lead to the higher adsorption capacity of Co^2+^ ions by the magnetic biosorbent. In aqueous solutions, −FeOH groups are present on iron oxide surfaces, and depending on the solution pH, they protonate or deprotonate to FeOH_2_^+^ and FeO^−^ [40]. With the increase in the solution pH, FeO^−^ increased, facilitating the removal of Co^2+^ by electrostatic adsorption [10,25]. Motl et al. [36] revealed that cobalt sorption by commercial magnetite proceeded via the ion-exchange of Co^2+^ and/or Co(OH)^+^ for the H^+^ cations of surface ionogenic –FeOH groups. However, due to the complexity of the biomaterials, the above-mentioned mechanisms are acting simultaneously, to varying degrees, depending on the biosorbent surface ionization and the solution chemistry [31].

**Figure 9:**
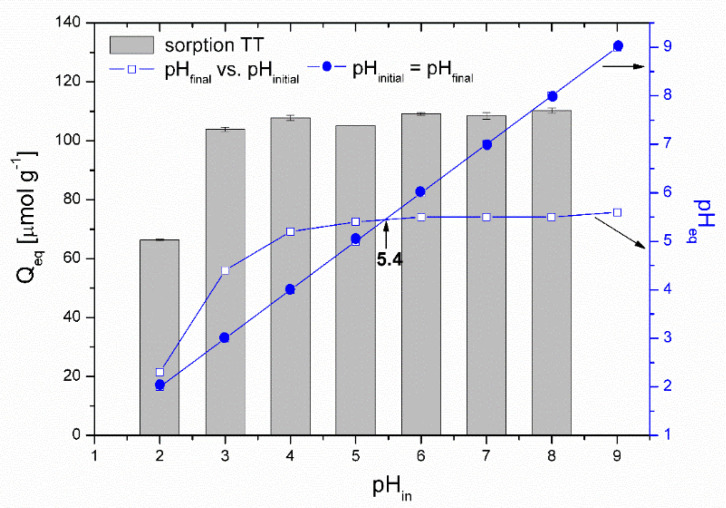
pH_pzc_ determination and the effect of initial pH on the adsorption of TT (313 µmol L^−1^) by the magnetically responsive biosorbent (2.5 g L^−1^; T = 22 °C).

**Figure 10:**
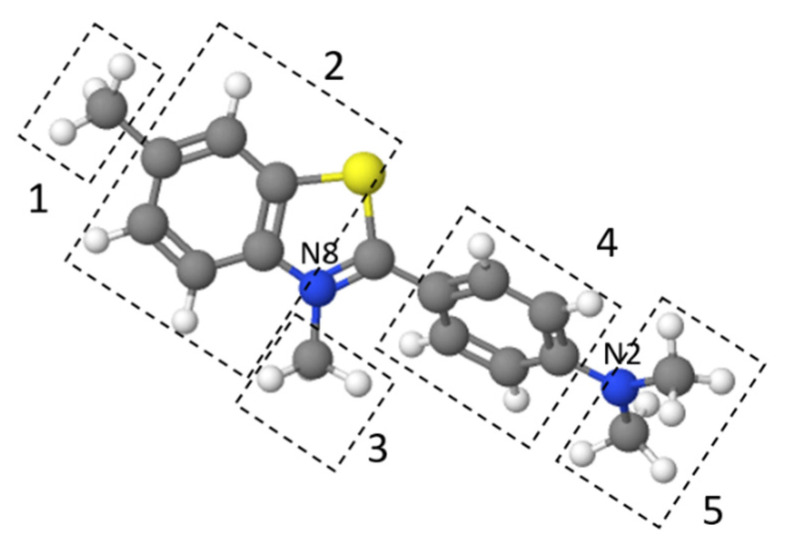
The structure of thioflavin T optimized using MolView. 1 and 3: methyl groups, 2: benzothiazole ring, 4: benzyl ring, and 5: dimethylamino group.

Figure 9 presents the effect of pH on TT adsorption. The thioflavin T molecule includes three structural fragments: (1) a benzyl ring, (2) a benzothiazole ring, and (3) the dimethylamino group, as shown in Figure 10. In general, the TT molecule has a positive charge (+1e) on the N atom (N8) that is non-uniformly distributed between the molecule fragments [41], indicating that the ionic nature of TT could play a role in retaining the TT species on the biosorbent surface. At low pH values (pH 2.0), the N2 atom of the dimethylamino group is protonated, and the electrostatic repulsion between the positively charged biosorbent surface and TT molecules causes a decrease of adsorption capacity. At higher pH values (pH 4.0 to 8.0), TT adsorbed on the surface and balanced the negative charge of the magnetic biosorbent. During the biosorption of TT at pH > pH_pzc_, the equilibrium solution pH decreased (Figure 9) due to the deprotonation of the surface acidic functional moieties accompanied by the release of H_3_O^+^ as the TT molecules were adsorbed. Similar to Co adsorption, the synergistic interaction with the –FeOH groups of iron oxides caused better TT adsorption than that of the unmodified biomass counterpart. Consequently, one of the possible mechanisms of TT biosorption by magnetically responsive *R. squarrousus* is the electrostatic attraction [29].

### 3.3. Magnetic Biosorbent Reusability and Stability

The appropriate handling of exhausted biosorbents is a limiting process influencing the economical efficiency and sustainability of biosorption technology. However, biosorbent reusability could effectively reduce the cost of industrial application and minimize the total amount of waste generation after the adsorption process [42]. Considering the results from preliminary desorption experiments (not shown), a magnetically separated biosorbent was regenerated using 0.1 M HCl (Co-loaded) and 0.1 M CH_3_COOH (TT-loaded) as efficient desorbents and then used in four successive adsorption–desorption cycles. 

**Figure 11:**
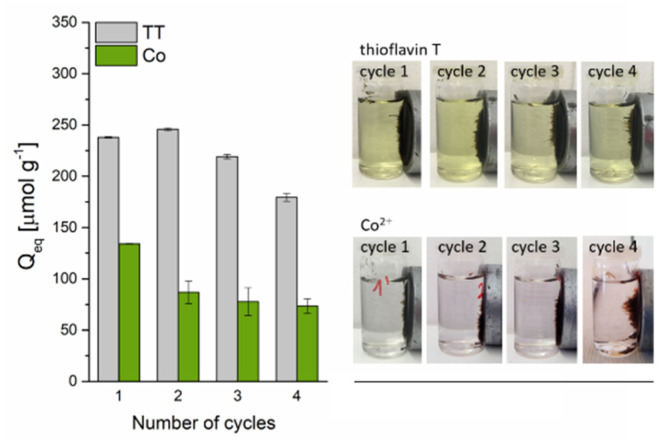
The reusability of magnetically responsive biosorbent (2.5 g L^−1^) for Co^2+^ (C_0_ = 1 mmol L^−1^ CoCl_2_) and TT (C_0_ = 0.63 mmol L^−1^) adsorption. Regeneration using 0.1 M HCl (Co-loaded) and 0.1 M CH_3_COOH (TT-loaded) at L/S = 0.8 L g^−1^.

The cyclic experiments (Figure 11) revealed that the TT adsorption capacity decreased slightly from 238.6 to 213.2 µmol g^−1^ after the third cycle and to 179.6 µmol g^−1^ after the fourth cycle, indicating a high adsorption ability and a good reusability of the magnetic biosorbent for TT removal. In contrast, the adsorption capacity of Co^2+^ on the biosorbent significantly dropped from 134.2 to 86.8 µmol g^−1^ after the first adsorption–desorption cycle. After the next three cycles, only a slight decrease to 73.4 µmol g^−1^ was observed. The markedly higher decrease in the adsorption capacity of Co^2+^ ions on the biosorbent during the adsorption–desorption cycles as compared to TT confirmed the different adsorption behavior. In addition, four adsorption–desorption cycles practically did not affect the magnetic separation of biosorbent (Figure 11), confirming the very good stability of microwave synthesized nanoparticles and microparticles of iron oxides impregnated on the biomass surface also in an acidic environment (0.1 M HCl and 0.1 M CH_3_COOH), which is in agreement with the study of Wang et al. [43]. Moreover, as was pointed out by Philben et al. [44], the physical and chemical structure of moss cell walls cause their high persistence in the environment, and this could also contribute to their notable biocomposite stability.

## 4. Conclusions

A magnetically responsive biocomposite from *R. squarrosus* was successfully prepared using microwave synthesized nanoparticles and microparticles of iron oxides for the efficient removal of Co^2+^ and TT from aqueous solutions. The biocomposite displays improved biosorption performance compared with their non-magnetic biomass counterpart. Obtained experimental data of both Co^2+^ and TT biosorption were well fitted with the pseudo-*n*-order kinetic model and Langmuir isotherm with a maximal adsorption capacity of 218 μmol g^−1^ for Co and 483 μmol g^−1^ for TT. Based on the EDX elemental mapping, besides the oxygen-containing functional groups of biomass biopolymers, synergistic interaction with the –FeOH groups of iron oxides also participates in Co^2+^ and TT adsorption. The cyclic experiments showed that the adsorption capacity of biocomposite kept 75% for TT and 55% for Co^2+^ after the fourth cycle, indicating a high adsorption ability and a good reusability of the magnetic biosorbent especially for TT removal. Considering the obtained results, the prepared magnetic biosorbent is superior to other adsorbents in terms of its simple preparation, efficient separation, reusable properties, and high stability, making it suitable for both dye and metal adsorption from contaminated effluents and wastewater streams.

## Figures and Tables

**Table 1:** Kinetic parameters of Co^2+^ and thioflavin T (TT) adsorption for magnetically responsive biosorbent calculated from a pseudo-*n*-order model using nonlinear regression analysis.

	C_0_ (µmol L^−1^)	*Q_e cal_* (µmol g^−1^)	*k_n_* (min^−1^) (mg g^−1^)^1−n^	*n*	*R* (µmol g^−1^ min^−1^)	*R* ^2^	*Q_e exp_* (µmol g^−1^)
Co^2+^	500	87.6 ± 2.5	0.0010 ± 0.0002	2.41	48.0	0.995	88.5
	1000	131.9 ± 4.3	0.0013 ± 0.0003	2.20	60.1	0.991	135.8
TT	313	106.7 ± 0.7	0.0448 ± 00235	1.51	51.7	0.999	105.0
	626	235.8 ± 2.3	0.0017 ± 0.0005	2.08	146.3	0.998	228.3

**Table 2:** Parameters of Langmuir and Freundlich isotherms for Co^2+^ and thioflavin T adsorption by native and magnetically responsive biosorbents calculated using nonlinear regression analysis.

Sorbent		Langmuir			Freundlich		
		*Q_max_* [µmol g^−1^]	*b* [L µmol^−1^]	*R* ^2^	*K* [µmol g^−1^ (L µmol^−1^)^1/n^]	1*/n*	*R* ^2^
Magnetic biomass	Co^2+^	218 ± 14	0.0025 ± 0.0006	0.980	12.35 ± 5.16	0.35 ± 0.06	0.956
	TT	483 ± 35	0.0072 ± 0.0015	0.961	39.78 ± 16.46	0.34 ± 0.07	0.858
Native biomass	Co^2+^	208 ± 3	0.008 ± 0.001	0.997	32.2 ± 16.5	0.24 ± 0.07	0.856
	TT	395 ± 10	0.06 ± 0.01	0.906	99.7 ± 5.5	0.19 ± 0.01	0.983

**Table 3:** The maximal adsorption capacities *Q_max_* of for Co^2+^ and TT by different sorbents determined from the Langmuir model.

Sorbent	*Q_max Co_* (µmol g^−1^)	*Q_max_*_TT_ (µmol g^−1^)	pH	T (°C)	Reference
*Vesicularia dubyana* moss	-	373	6.0	25	Pipíška et al. [29]
Hop leaf biomass	-	243	6.0	25	Partelová et al. [30]
*Rhytidiadelphus squarrosus*	208	395	6.0	25	Remenárová et al. [23] Remenárová et al. [31]
Modified montmorillonite	-	298	6.0	25	Shin [32]
*Fomitopsis carnea*	-	68.7	-	30	Maurya and Mittal [33]
ultrathin-shell boron nitride hollow spheres	-	479	-	-	Lian et al. [34]
Magnetic *R. squarrosus*	218	483	6.0	22	Present study
Fe_3_O_4_/bentonite nanocomposite	323	-	8.0	25	Hashemian et al. [35]
Magnetite	25	-	8.0	-	Motl et al. [36]
Green microalgae magnetic composite	2327	-	6.5	20	Zhong et al. [37]
*Ficus benghalensis*	97	-	5.0	25	Hymavathi and Prabhakar [38]
